# 

**Published:** 1897-10

**Authors:** 


### CONTENTS.

ORIGINAL ARTICLES: PAGE
Presentation of the Subject of State Control of Tuberculosis. By JOHN M. PARKER, D.V.S. . . . . 607
The Need of Veterinary Education in Medical Colleges. By E. P. NILES, V.S. . 615
Inhalation-Pneumonia. By W. L. WILLIAMS, V.S. and P. A FISH, D.V.S. . . 619

ABSTRACTS FROM FOREIGN JOURNALS:
Therapeutics of Chronic Otitis in the Dog . . . . . 627
Tubercular Synovitis and Metastatic Inflammation of the Joints . . . . 628
Tuberculous Leptomeningitis Basilar . . . . . 629
Infectious Material of the Aphtha Epidemic . . . . . 630
Infectious Diseases in Man . . . . . 630
Count Gattenberg, Member of the Lower Austrian District Committee, Celebrates the Twenty-fifth Anniversary of that Event . . . . . 630
The Etiology and Rational Treatment of Anasarca. By Dr. CADEAC . . . . 631
Equine Nasal Polypi, with Profuse Discharge . . . . . 631
Arthritis and Synovitis. By Dr. GUITTARD . . . . . 632
The Treatment of Pulmonary Emphysema in the Horse . . . . . 632

SELECTIONS . . . . . 633

REPORTS OF CASES:
Rupture of the Liver. By W. J. MARTIN, V.S. . . . . . 635
An Abnormal Condition of the Forelegs of a Colt. By H. S. PERLEY, V.S. . . 637
Four Cases of Parturient Apoplexy. By W. E. MCCRAY, V.S. . . . . 637
I. Tenotomy. II. Resection of Necrotic Parts of the Soft Tissues of the Frog for Nail-wound. By OTTO G. NOACK, V.S. . . . . . 638

EDITORIALS:
The Nashville Meeting . . . . . 641
Will it Prove a Wise Departure . . . . . 644

GLEANINGS . . . . . 646
CONTROL WORK . . . . . 647
ALWAYS TO YOUR INTEREST TO READ THESE SELECTIONS . . . 648
AMONG THE COLLEGES . . . . . 650

SOCIETY PROCEEDINGS:
U. S. V. M. A. The Story of Nashville—Pennsylvania State Association of Master Horseshoers—Veterinary Medical Association of the District of Columbia—U. S. Experiment Station Veterinarians—United States Veterinary Medical Association . . . . . 651–675

PERSONALS . . . . . 675
NEW INVENTIONS . . . . . 678

